# Portable nuclear magnetic resonance biosensor and assay for a highly sensitive and rapid detection of foodborne bacteria in complex matrices

**DOI:** 10.1186/s13036-017-0053-8

**Published:** 2017-03-27

**Authors:** Yilun Luo, Evangelyn C. Alocilja

**Affiliations:** Department of Biosystems and Agricultural Engineering, Michigan State University, East Lansing, MI 48824 USA

**Keywords:** Biosensors, Nuclear magnetic resonance, Nanotechnology, Magnetic nanoparticles, Magnetic separation

## Abstract

**Background:**

Nuclear magnetic resonance (NMR) technique is a powerful analytical tool in determining the presence of bacterial contaminants in complex biological samples. In this paper, a portable NMR-based (pNMR) biosensor and assay to detect the foodborne bacteria *Escherichia coli* O157:H7 is reported. It uses antibody-functionalized polymer-coated magnetic nanoparticles as proximity biomarker of the bacteria which accelerates NMR resonance signal decay.

**Results:**

The pNMR biosensor operates at 0.47 Tesla of magnetic strength and consists of a high-power pulsed RF transmitter and an ultra-low noise sensing circuitry capable of detecting weak NMR signal at 0.1 μV. The pNMR biosensor assay and sensing mechanism is used in detecting *E. coli* O157:H7 bacteria in drinking water and milk samples. Experimental results demonstrate that by adding a filtration step in the assay, the pNMR biosensor is able to detect *E. coli* O157:H7 as low as 76 CFU/mL in water samples and as low as 92 CFU/mL in milk samples in about one min.

**Conclusion:**

The pNMR biosensor assay and sensing system is innovative for foodborne bacterial detection in food matrices. The lowest detection level for *E. coli* O157:H7 in water and milk samples is essentially 10^1^ CFU/mL. Although the linear range of detection is only from 10^1^ to 10^4^ CFU/mL, the wider detection range spans from 10^1^ CFU/mL to 10^7^ CFU/mL. Existing pNMR biosensors have detection limits at 10^3^-10^4^ CFU/mL only. The detection technique can be extended to other microbial or viral organisms by merely changing the specificity of the antibodies. Besides food safety, the pNMR biosensor described in this paper has potential to be applied as a rapid detection device in biodefense and healthcare diagnostic applications.

## Background

### Nuclear magnetic resonance

Nuclear magnetic resonance (NMR) is a physical phenomenon where certain atomic nuclei absorb and re-emit electromagnetic radiation when placed in a magnetic field. NMR technique exploits this phenomenon to analyze the magnetic properties of atomic nuclei and determine chemical properties of bio-molecules. Since its signal is able to penetrate turbid raw samples, NMR techniques have wide applications in non-destructive food and biomedical diagnoses, which can simplify sample preparation processes and save analysis time [1]. During the early stages of development, NMR analyses were performed in laboratories by placing the sample of interest inside a strong stationary magnet [2]. Although the configuration produced accurate results, the instruments were rather large, heavy, not portable, and expensive [2, 3]. This has made the NMR technique less practical and less useful for on-site applications. Continuous development however resulted in the advancement of portable NMR (pNMR) sensor hardware and detection performance. pNMR sensors use magnetic nanoparticles and microparticles as proximity sensors to amplify molecular interactions [4]. They are based on the reversible self-assembly of dispersed magnetic particles into stable nano-assemblies [4]. When a few magnetic nanoparticles bind their intended molecular target through affinity ligands, they form magnetic clusters which leads to a corresponding decrease in the bulk spin-spin relaxation time (*T*_2_) of the surrounding water molecules [5]. Furthermore, measurements can be performed in turbid samples with minimal sample preparation and the sensing method is faster than those with surface-based techniques relying on molecular diffusion of targets to the sensing elements. These advantages render the proximity assay ideal for fast, simple and high-throughput sensing operations, especially in miniaturized device format. This class of sensors is more flexible, smaller in size, and suited for on-site and field-based measurements. They are also cheaper to develop and less costly to maintain as compared to bulky conventional NMR instruments.

A portable NMR system was reported in 2000 for a non-invasive spin-echo imaging of living plants in their natural environment [6]. Recent advances in micro-fabrication technologies have accelerated the development of palm-sized NMR and high-throughput NMR spectrometers. The palm-sized NMR used integrated circuit technique and was tested in detecting biological molecules and cancer cells by measuring the sample’s NMR relaxation time which was proportional to the immunological clustering of magnetic nanoparticles [5]. The reported sensitivity for cancer cell detection was in the range of 10^3^-10^4^ cells/mL [7]. Other portable NMR developments include various designs [2–4, 6, 8, 9]. The development of high-throughput NMR spectrometers using complementary metal oxide semiconductor (CMOS) technology consisted of an array of high sensitivity micro-coils integrated with interfacing radio-frequency circuits on the same chip [10]. A micro-nuclear magnetic resonance (μNMR) relaxometer miniaturized to palm-size and electronically automated for multi-step and multi-sample chemical/biological diagnosis was also developed [11]. The μNMR relaxometer integrated microfluidic and microelectronic technologies to enable the association between the droplet management and μNMR assay inside a portable sub-Tesla magnet. Targets in unprocessed biological samples, captured by specific probe-decorated magnetic nanoparticles, were sequentially quantified by their spin–spin relaxation time (T_2_) via multiplexed μNMR screening. An NMR that can fit a 2 mm by 2 mm silicon chip was also reported [9].

### Bacterial contamination

Food safety is a major concern with so many microbial contaminations in recent years. *Escherichia coli* O157:H7 is one of the major bacterial pathogens that has caused foodborne illness outbreaks in the US and around the world. In 2009, *E. coli* food contamination in beef caused a foodborne illness outbreak across 8 US states for a total of 26 identified cases and 2 deaths, a recall of more than half a million pounds of ground beef products, and caused the Centers for Disease Control and Prevention (CDC) to issue a health alert [12, 13]. As recent as September 2016, *E. coli* O157:H7 contaminated beef caused a multi-state illness outbreak [14]. The organism has also been implicated in various other types of food, such as spinach, cookie dough, cheese, sausage, and hazelnut. The aggregated annual cost of foodborne illness in the US alone is estimated at $77.7 billion [15].

The detection of *E. coli* O157:H7 is time consuming and requires complex instruments and extensive training. The procedure involves several steps including selective enrichment, filtration, incubation, confirmation, biochemical, and serological techniques [16]. Polymerase chain reaction (PCR) based detection assays are sensitive and accurate however, they require complex sample preparation steps, such as DNA extraction and amplification, which increase additional diagnostic time [17, 18]. Fast detection methods have been reported based on immunological detection. However, most of these diagnostic systems have sensitivity limit greater than 10^2^ CFU/mL, detection time of greater than 1 h, and are not applicable for on-field applications [19]. In order to minimize the spread of infection and costly product recall, a rapid, sensitive, and portable detection of *E. coli* O157:H7 is essential in the food supply and healthcare applications.

Learning from the basic NMR design by Fukushima and Roeder [20], we developed a portable NMR-based biosensor and assay for the rapid and sensitive detection of foodborne pathogens. The novelty of our pNMR biosensor includes filtration assay, use of inexpensive NMR probe, and RF transceiver for microbial detection in complex matrices. The proximity biomarker uses an antibody-functionalized magnetic nanoparticles (Ab-MNP). The system is low cost, small in size, and highly portable for foodborne pathogen detection.

## Methods

### Design of pNMR biosensor

**Fig. 1:**
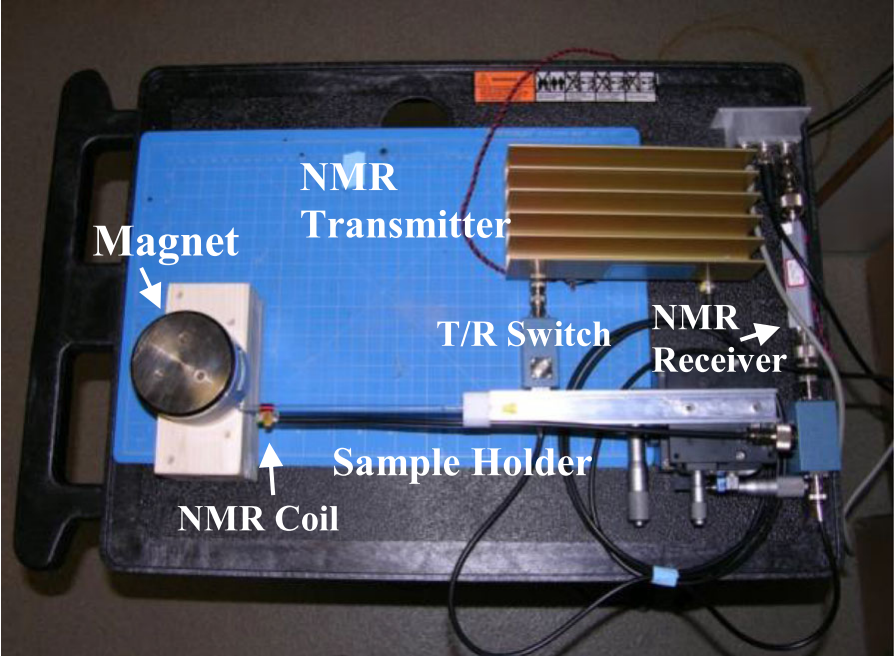
System prototype of portable NMR

Based on the principles of generic NMR [20], a pNMR biosensor was designed consisting of a proton NMR probe, high power and high sensitivity transmitter and receiver, FPGA based pulse controller, and communication interface. The system utilized a 0.49 Tesla permanent magnet in a compact size of Φ80 x H55 mm (PM-1055, Metrolab Instruments Inc.). A gauss meter was used to calibrate the magnetic field strength and determine its most homogeneous region. A solenoid coil with size of Φ5 x L5 mm was fabricated in the lab and matching networks using high-Q capacitor trimmers were designed to achieve an optimal signal-to-noise ratio (SNR) [20]. The NMR transmitter was capable of generating 19.9 Mhz signal at 20 Watts, enough to excite water nuclei resonance spin inside the NMR probe. A transmit/receive (T/R) switch was designed using high speed crossed diodes and quarter wavelength transmission line to protect low noise amplifier during high power excitation and block the noise from LPA during receiving the signal. The NMR receiver had high amplification gain and very low noise. It was capable of detecting a signal around 0.1 μV, which was reemitted from the excited nuclei spinning in resonance. With excellent concurrent calculation capability and high integration, an embedded system was designed in FPGA using multilayer state machine to receive commands and display via HyperTerminal, control NMR transmitter frequency and amplification gain, and provide precise control to generate versatile NMR pulse sequence. A prototype of the portable NMR system is shown in Fig. 1. The magnet holder, NMR probe holder, and gauss meter probe holder were designed using aluminum and wood, which does not interfere with the magnet’s property. An X-Y-Z precision linear positioner was used to determine the most homogeneous region of the magnet and adjust NMR sample position in order to optimize NMR sensitivity. The prototype system was built using low cost amplifiers, 50B power amplifier (Henry Radio), and AU-1467 linear amplifier (Miteq Inc.). The overall size of the prototype was 32 cm x 24 cm x 14 cm. The system can be integrated in a mini personal computer enclosure, 20 cm x 18 cm x 8 cm for better portability and electromagnetic compatibility (EMC) performance. Low cost commercially available 5-mm NMR tube was used as test sample holder, and was reusable after acetone washing. The sample volume of the prototype system was 100 μL. It could be further reduced by using smaller 20 μL NMR tubes.

### Test pathogen and antibodies

*E. coli* O157:H7 was obtained from the collection of the Nano-Biosensors Laboratory at Michigan State University. *E. coli* O157:H7 test strains were inoculated using sterile loop into 10 mL of Tryptic soy nutrient broth from Difco Laboratories (Detroit, MI) and incubated for 24 h at 37 °C to make a stock culture. The stock culture was then serially diluted in 0.1% peptone water in logarithmic scale to obtain different concentrations. All of the experiments were performed in a certified Biological Safety Level II laboratory. The antibody used for NMR biosensor was purified mouse anti-*E. coli* O157:H7 monoclonal antibody (Meridian Life Science, Inc. Saco, ME).

### Antibody functionalization of magnetic nanoparticles

Magnetic nanoparticles (MNPs) were synthesized from aniline monomer and gamma iron (III) oxide (γ-Fe_2_O_3_) nanoparticles with weight ratio of 0.6:1 using a sol-gel chemical solution deposition method according to Pal et al. [21]. The MNPs were filtered using 0.22 μm syringe filter (Millipore, MA, USA) to remove large particles. Pal et al. [21] had previously shown that the different ratios of iron oxide to aniline (1:0, 1:0.1, 1:0.4, 1:0.6, 1:0.8) resulted in various coercivity, retentivity, saturation magnetization, and temperature conductivity values. Electron diffraction micrographs of these ratios were also studied. The ratio of 1:0.6 was shown to be a better material in terms of these parameters*.*

To conjugate the antibodies to MNPs (Ab-MNP), 2.5 mg of MNPs were added into a 3 mL test tube. Then, 150 μL of 0.1 M phosphate buffer solution (PBS) was added to the test tube and sonicated for 15 min to evenly disperse the particles. Then, 100 μL of 2.5 mg/mL monoclonal anti *E. coli* O157:H7 antibody in PBS was added to the test tube and incubated in a hybridization oven at 37 °C for 1 h [22]. The sample was rotated in the oven at 30 revolutions per minute (rpm) to facilitate antibody functionalization. The incubated solution was purified by magnetic separation process for three times using 0.01 M Tris buffer with 0.01% casein solution, and finally suspended in 2.5 mL of 0.01 M PBS. This prepared Ab-MNP solution was stored at 4 °C in the refrigerator until needed.

### Magnetic pathogen interaction and filtration assay

**Fig. 2:**
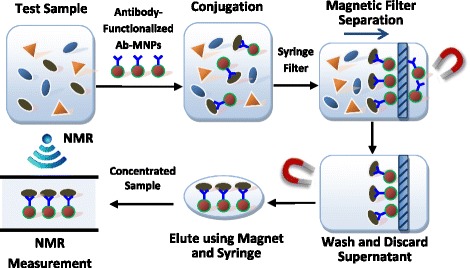
Working principle of the NMR based biosensor for pathogen detection

Fifty μL of Ab-MNP conjugates and 50 μL of pathogen sample dilution were mixed in 400 μL of 0.01 M PBS. For negative control (blank sample), 50 μl of 0.1% (w/v) peptone water was used instead of pathogen sample dilution. All mixtures were incubated at 25 °C at 60 rpm for 30 min for MNP-pathogen conjugation. In order to enhance NMR sensitivity, the incubated solution was filtered using a 0.45 μm syringe filter (Millipore, MA, USA) to remove impurities and unbound Ab-MNPs. The syringe filter’s pore size was determined to be as large as possible (0.45 μm) in order to ensure that all the unbound particles could flow through but would keep all the target bacteria. After the washing process using 0.01 M PBS for three times, the syringe filter was back flushed using 5 mL of 0.01 M PBS to release the MNP-pathogen conjugates for use as sample in the NMR biosensor. During the separation, wash, and back-flush process, a magnet was used to facilitate the separation of unbound Ab-MNPs, hold the MNP-pathogen conjugates during washing, and help elute the MNP-pathogen conjugates. The filter based magnetic separation process is illustrated in Fig. 2.

### Sensor architecture and detection principle

**Fig. 3:**
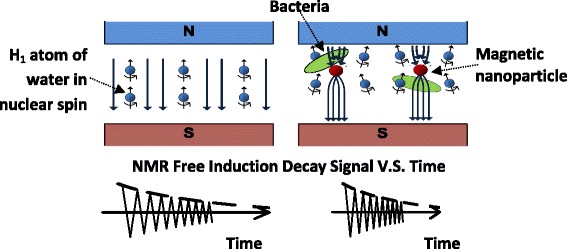
Magnetic nanoparticles as proximity biomarker for NMR detection

After the filtration assay, the interference of unbound Ab-MNPs was effectively reduced, and MNPs in the eluted solution were proportional to the pathogen concentration. The MNP induced spatial and temporal disturbance in the homogeneity and strength of the local magnetic field (Fig. 3). Due to the high surface area to volume ratio, this disturbance introduced precession frequency variations in millions of protons of the surrounding water molecules, which accelerated the decay of the spin system’s phase coherence. It was earlier shown that the magnetic particle’s concentration had a linear relationship to the water proton’s spin-spin relaxation time, T_2_. Therefore, the concentration of target pathogen in the test solution could be measured from the T_2_ signal using the pNMR biosensor.

### Detection and data analysis

The pNMR biosensor signal was measured using a digital oscilloscope Model Agilent DSO1024A (Agilent Technologies, Santa Clara, CA) that was connected to the pNMR’s signal output using BNC cable. For the biosensor measurement, a volume of 100 μL of the test solution was placed in the NMR tube. The pNMR spin-echo relaxation signal was recorded by the oscilloscope, which was controlled by FPGA synchronization signal. The whole process of pNMR relaxation was less than 1 min. For data analysis, a minimum of three replications were performed for each experiment. All measurements were calibrated using a control sample without the pathogen. Standard deviations and mean values for the data were calculated using Excel.

## Results and discussion

**Fig. 4:**
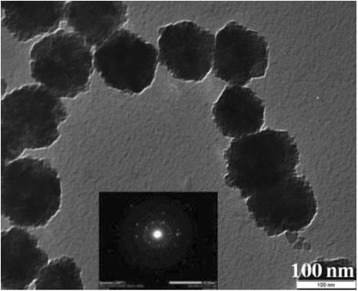
TEM image and electron diffraction (inset) image of the MNP

The TEM image of the MNP (Fig. 4) demonstrates that the particles are spherical in shape with a uniform average size of 80 nm and a total distribution of 50 to 100 nm [21]. The crystalline nature of MNPs are confirmed by the electron diffraction rings as shown in Fig. 4 (inset). As shown earlier [21], the saturation magnetization, *M*_*S*_, of the MNP (1:0.6) was obtained by M-H hysteresis measurement, and was found to be 38 emu/g at room temperature. The measured coercivity (*H*_*C*_) of MNP was 180 Oe, the same as the unmodified Fe_2_O_3_ NPs, indicating that the anisotropy energy of the MNPs was not affected by the polyaniline shell.

The antibodies were conjugated onto MNP by physical non-covalent adsorption. The Ab-MNP conjugation is mainly formed by electrostatic interaction between the positively charged polyaniline surface and the negatively charged Fc portion of the antibodies, along with other factors, such as van der Waals interaction, electrostatic interaction, hydrophobic effect and hydrogen bonding [21, 23]. The successful functionalization was earlier confirmed by spectrophotometric studies [21]. From UV-VIS scanning spectrophotometer measurement, pure antibody solution showed a characteristic peak of protein molecules at 280 nm. After the functionalization process and three times of magnetic separation, the supernatant solution showed no peak at 280 nm, demonstrating that antibodies were effectively conjugated onto the MNPs.

**Fig. 5:**
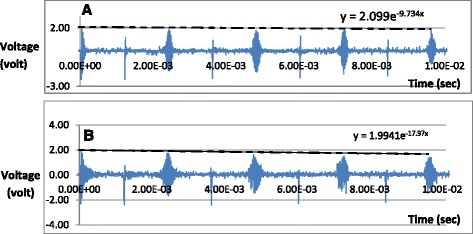
Typical NMR biosensor relaxation signal: **a** sample with no bacteria (blank, control); **b** sample spiked with *E. coli O157:H7* bacteria

The NMR biosensor measures the spin-spin relaxation time (T_2_) of water proton in proximity to the magnetic nanoparticles in the test sample. The spin-spin relaxation consists of echo signals with exponential decay characteristics and can be expressed mathematically as shown in Eq. 1.

1 $$ M(t)={M}_0\ {e}^{-\frac{t}{T_2}} $$

where *M(t)* is the nuclear spin magnetization vector at any point in time t, M_0_ is the initial nuclear spin magnetization vector, and T_2_ is the spin-spin relaxation time constant. The relaxation time T_2_ is calculated from the curve fitting of the signal envelope of the NMR decay. For example, two NMR relaxation signals are presented in Fig. 5. Figure 5a is the signal envelope of a sample with no bacteria (blank) while Fig. 5b is the signal envelope of a sample spiked with the bacteria *E. coli* O157:H7. In this particular experiment, the spiked sample had a plate count bacterial concentration of 226 colony forming units of bacteria per milliliter (CFU/mL). The curve fitting is shown as dashed lines in Fig. 5 and the corresponding equations are shown above the dashed lines. The envelope curve fitting in Fig. 5a resulted mathematically in the equation:

2 $$ \mathrm{y}=2.099\ {\mathrm{e}}^{\hbox{-} 9.734\mathrm{x}} $$

Using Eq. 1, the time constant 1/T_2_ is 9.734. By expansion, T_2_ is equal to 1.027 s or 102.7 ms. For the spiked sample (Fig. 4b), the envelope curve fitting resulted in the equation:

3 $$ \mathrm{y}=1.9941\ {\mathrm{e}}^{\hbox{-} 17.97\mathrm{x}} $$

In this case, 1/T_2_ is equal to 17.97 resulting in T_2_ being equal to 0.0556 s or 55.6 ms. T_2_ of the spiked sample is 54.1% (55.6/102.7) shorter than that of the blank sample. These data show that T_2_ decays faster in samples with bacteria than in samples with no bacteria due to the formation of magnetic clusters around the bacterial cell walls and correspondingly decreasing the bulk spin-spin relaxation time of the surrounding water molecules.

**Fig. 6:**
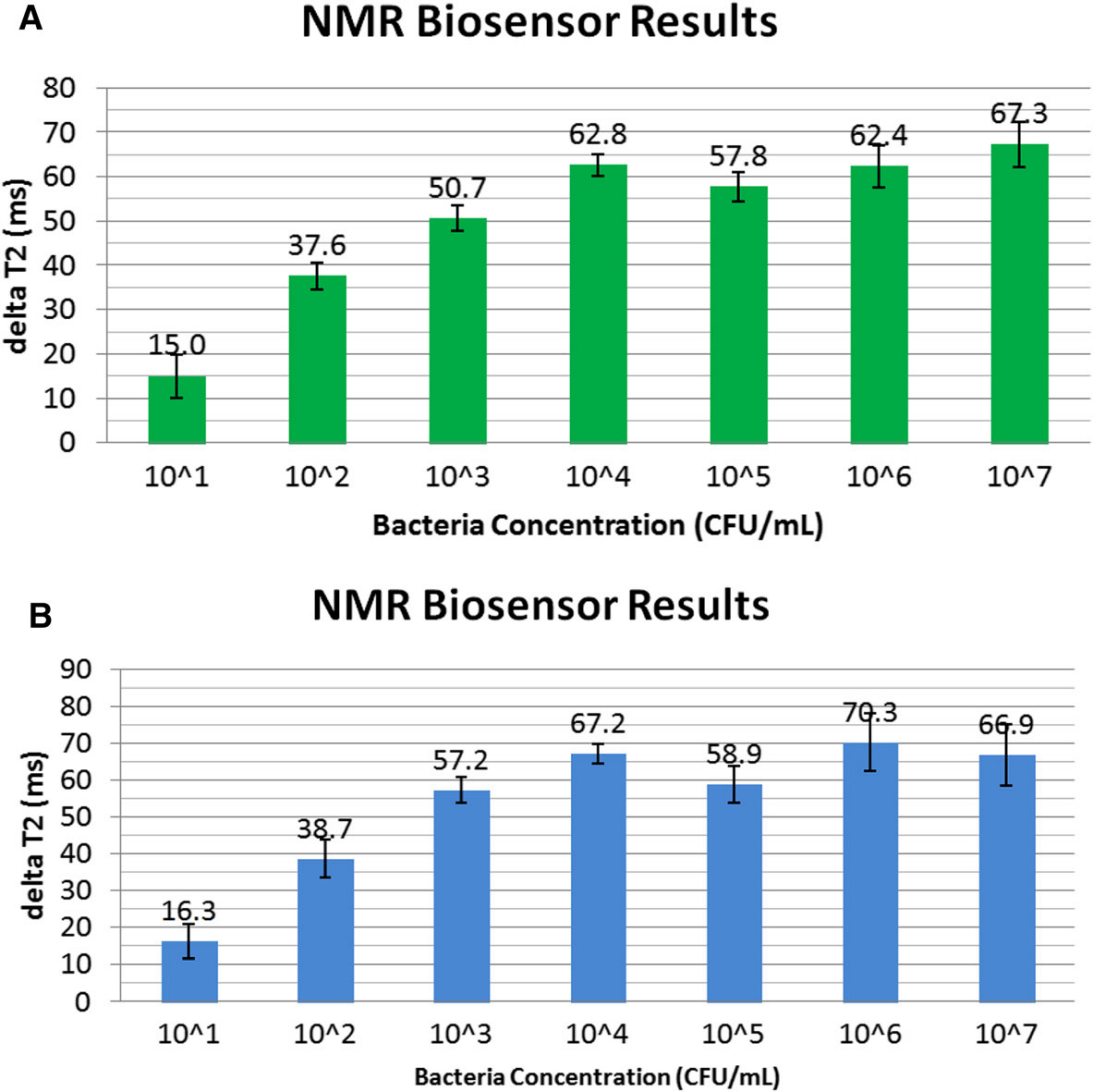
The MNP based NMR biosensor’s measurement of relaxation time change, delta T_2_, in ms of (**a**) water and (**b**) whole milk samples contaminated with *E. coli* O157:H7 bacteria. NMR = nuclear magnetic resonance; ms = millisecond

Drinking water and whole milk were used in the experiment to represent food samples. Water and milk were artificially contaminated with *E. coli* O157:H7 ranging in concentration from 10^1^ CFU/mL to 10^7^ CFU/mL. Filtration and detection followed the same procedure as described above. Figure 6a and b show the NMR result, delta T_2_ measurements of water and whole milk samples using the pNMR biosensor. The envelope curve fitting was generated for each experiment and T_2_ was calculated as shown above. The average and variance of the delta T_2_ values (T_2 Control_ − T_2 Sample_) for three replicates were plotted for the control and contaminated samples. Figure 6a and b show that the relaxation times of all the contaminated samples are shorter than that of the control. In water samples (Fig. 6a), the delta T_2_ increases linearly from 10^1^ up to 10^4^ CFU/mL. The increased relaxation time difference between the control and contaminated samples supports the formation of magnetic nanoparticles bound to the target bacteria, indicating a change in their nearby magnetic field and affecting the nuclear spin of hydrogen atoms in surrounding water molecules. The pNMR signal does not change when the bacterial concentration is 10^5^ CFU/mL and higher. This effect could be attributed to a number of factors. First, in high bacterial concentrations, the outer bacteria could form a blocking shell to the magnetic core and hence reduce the MNP’s influence on the NMR relaxation signal. A second factor could be that there is not enough Ab-MNP to capture all the bacteria in the sample, similar to a saturated effect. In fact, data show that the NMR signals for bacterial concentration 10^5^-10^7^ CFU/mL are not much different from the NMR signal of 10^4^ CFU/mL.

The NMR biosensor signal for whole milk is comparable with that to water. The filtration assay has basically cleaned-up the matrix. Overall, the lowest detection for milk and water are in the order of 10^1^ CFU/mL or specifically, 76 CFU/mL for water and 92 CFU/mL for milk. Bacteria can be detected from 10^1^ CFU/mL to 10^7^ CFU/mL. The wide detection range is highly relevant to food safety because certain organisms have varied infectious doses. For example, studies have shown that the infectious dose for some *Shigella* spp. is less than 10 organisms while the infectious dose for toxigenic *V. cholera* is 10^4^ organisms [24]*.* The infective dose for Salmonella is 10^3^ organisms [25].

Results from the NMR biosensor show that the sensitivity is better than those in the existing literature. A chip-NMR biosensor designed by Lee et al. has a detection sensitivity of only 10^3^ CFU/mL for *Staphylococcus aureus* [6]. This level of detection limit is consistent with our results if filtration is not applied (10^3^ CFU/mL of *E. coli* O157:H7, data not shown).

## Conclusion

This paper has described a portable NMR biosensor that has been applied to detect *E. coli* O157:H7 in food matrices. The bacteria are labeled with the MNP through antibody-pathogen interaction. The detection of the biosensor system is fast, which includes a filtration assay for 20-30 min followed by signal detection of 1 min. The average detection limit for water and milk is 84 CFU/mL, much lower than those reported in the literature. The linear range of detection is from 10^1^ to 10^4^ CFU/mL while the detection range spans from 10^1^ CFU/mL to 10^7^ CFU/mL. The detection technique can be extended to other microbial or viral organisms with a change in the specificity of the antibodies in the Ab-MNP system. Besides food safety, the NMR biosensor described in this paper has potential to be applied as a rapid detection device in biodefense and health diagnostic applications.
